# Insights into Genomic Patterns of Homozygosity in the Endangered Dülmen Wild Horse Population

**DOI:** 10.3390/genes16091054

**Published:** 2025-09-08

**Authors:** Silke Duderstadt, Ottmar Distl

**Affiliations:** Institute of Animal Breeding and Genetics, University of Veterinary Medicine Hannover (Foundation), 30559 Hannover, Germany; silke.duderstadt@tiho-hannover.de

**Keywords:** genetic diversity, genomic selection, effective population size, ROH islands, selection signatures, inbreeding depression

## Abstract

Background/Objectives: Dülmen wild horses are kept in a fenced wooden and marsh area around Dülmen in Westphalia, Germany, since 1856. Previous analyses supported early genetic divergence from other domesticated horse populations and the Przewalski horse. Therefore, the objective of this study was to evaluate genetic diversity using high-density genomic data. Methods: We collected 337 one-year-old male Dülmen wild horses, captured at 12 annual auctions, for genotyping on the Illumina GGP Equine Plus Beadchip. All analyses were performed for 63,123 autosomal SNPs. Results: On average, each horse had 27.96 ROH with an average length of 8.237 Mb, resulting in an average genomic inbreeding coefficient F_ROH_ of 0.107. ROH with a length of 2–4 Mb were most frequent, and the next frequent ROH fall into the length categories of 4–8 and 8–16 Mb. The effective population size (N_e_) steadily decreased in the last 100 generations by 4.57 individuals per generation from 498 to 41. We identified 10 ROH islands on equine chromosomes 1, 4, 5, 7, 9, and 10. Only one ROH island on ECA 1 was shared by 45% of the horses. Overrepresented genes of ROH islands were associated with glycerophospholipid catabolism through *phospholipase A2* genes, skeletal muscle contraction (*TNNI3*, *TNNT1*), synapse activity and structure (*CTTNBP2*), regulation of inflammatory response (*NLRP* genes), and *zinc finger protein* genes, which are involved in many cellular processes and may also act as tumor suppressors and oncogenes. Conclusions: This study highlights the development of genomic inbreeding and shows the importance of the stallions selected for breeding on the genetic diversity of the Dülmen wild horses. The results of this study should be used to develop strategies to slow down increase in inbreeding and prevent transmitting unfavorable alleles from the stallions to the next generation.

## 1. Introduction

Dülmen wild horses in the region around Dülmen in Westphalia, Germany, date back to 1316 [1]. In the Middle Ages, these horses ranged in the wooden and marsh area in small herds. Since 1856, these wild horses have been fenced in the Merfelder Bruch near Dülmen in Germany by the Dukes of Croÿ and have been managed as an independent population to date. All decisions concerning this herd have been made by the Dukes of Croÿ for around 180 years. The female population sustained itself without crossbreeding with other domesticated horse breeds since 1856 [1]. Young males are annually removed from the herd and sold at yearly auctions. Stallions are either from the Dülmen wild horse population or come from other horse populations [1,2,3]. Registering of foals born and mares has not been introduced because all human interactions with the horses should be limited to a minimum. The Dukes of Croÿ aim to preserve the Dülmen wild horses as a free-roaming herd in their natural harsh environment. The survival of the horses depends mainly on how long they can withstand these natural conditions throughout the year. The Dukes of Croÿ select 2–3 stallions for each breeding season. The covering period for stallions is approximately 3–6 weeks in April and May. This short period during which the stallions can breed helps keep the size of the female herd at around 400 animals.

Pedigree data as in livestock populations are not available for the Dülmen wild horses; therefore, genetic diversity and size of inbreeding can only be estimated using genomic data based on microsatellites [2,3,4,5,6,7] or genome-wide SNP arrays [8]. Prior to the genomics era, pedigree-based inbreeding measures were widely used [8]. Microsatellites were the first molecular markers, particularly useful for paternity testing and studies on genetic diversity due to their high information content before SNP arrays became available [2,3,4,5,6,7]. Along with the availability of SNP genotypes for genomic evaluations in livestock, genome-wide SNP data have significantly improved the precision for kinships and inbreeding estimates and allowed the identification of lethal recessive mutations [8]. In addition, region-specific segments with low heterozygosity could be identified and more effectively used to manage conservation of endangered breeds [8]. Arrays with evenly genome-wide distributed SNPs are well suited to capturing runs of homozygosity (ROH) and the degree of genomic inbreeding [8,9,10,11,12,13,14,15,16,17,18,19,20,21,22,23,24,25,26,27]. In addition, the classification of ROH by their lengths allows us to infer the timing of inbreeding events, which informs management urgency. Furthermore, determination of ROH islands and consensus ROH, which are shared by a certain proportion of the members of a population, may be useful in detecting genomic regions associated with signatures of selection driven by a few loci or even one locus [9,10,11,12,13,14,15,16,17,18,19,20,21,22,23,24,25,26,27].

In horses, signatures of selection for coat color [9,22,23], body size [9,14,19,21,22,23,24], performance [14,25], disease resistance [21], immune system [24,27], and fertility [23,24,25,26,27] have been detected using ROH.

In previous reports, we used a highly informative set of autosomal microsatellites to explore the genetic diversity of Dülmen wild horses and to show they can be distinguished from other domesticated horse populations and classified as a self-standing population [2]. The neighbor-joining dendrogram of the Nei’s distance, with the Przewalski’s horses as the outgroup, showed a separate cluster for Dülmen wild horses, along with Dülmen, Liebenthal, and Polish Konik horses. This cluster split off before the common node for the branches of the other horse populations, including Sorraia, Icelandic, Exmoor, Friesian, Arabian, Hanoverian, and German coldblood breeds. Bayesian clustering also supported the early divergence of the Dülmen wild horses from other domesticated horse breeds and in addition, from Przewalski horses. Furthermore, we highlighted the contributions of the stallions to the genetic diversity and subclusters of the herd as well as the genetic distances between the paternal progeny groups [3]. This study highlighted the genetic contributions of the stallions to their male offspring.

In the present study, we supplemented the samples with additional young male Dülmen wild horses from the birth cohorts of 2015–2023 and genotyped 337 male Dülmen wild horses on a high-density SNP array, including 12 birth cohorts from the years 2012–2023. The present study should inform the genomic patterns of ROH and the genomic inbreeding of the Dülmen wild horses. Therefore, our objective was to evaluate the distribution of ROH and genomic inbreeding in this wild horse population (1), develop an effective populations size (2), search for ROH islands and explore these regions for overrepresented genes (3), analyze the development of genomic inbreeding by birth years and their temporal origins using ROH segments with different lengths (4), and, in addition, test the influence of the stallions on genomic inbreeding of their progeny (5) and on their seasonal breeding success rates per season (6).

## 2. Materials and Methods

### 2.1. Ethical Approval

The study was conducted in accordance with the guidelines of the Declaration of Helsinki and approved by the Institutional Review Board of the University of Veterinary Medicine Hannover (Foundation) and the state veterinary office from German Federal State for North Rhine-Westphalia (registration number 8.84-02.05.20.12.066, on 17 April 2012, 7 May 2015, 15 May 2018, and 11 May 2021). We adhered to the European Union’s guidelines for the care and handling of animals and good veterinary practice when sampling was performed.

### 2.2. Sample Collection and Genotype Data

Hair root samples from the manes (approximately 150–200 hair roots) were taken from 337 male Dülmen wild horses at the annual auctions in the Merfelder Bruch from 12 consecutive years. All samples were stored in sealable plastic bags at −20°, immediately after collection. Each year, at the end of May, all one-year-old colts are captured and sold at the yearly auction. Sampling was performed with the permission of the Duke of Croÿ. The foals were sired by 14 different Dülmen wild horse stallions, which originated from the herd in the Merfelder Bruch. Dülmen wild horse mares were sired by two stallions, except for three seasons (2012, 2017, 2018), when they were sired by three stallions. A rough estimate of mares giving birth to foals may be between 60 and 100. The breeding period per stallion lasts from 3 to 6 weeks per season. A survey on the number of the sampled and genotyped male horses by year of siring, birth, and auction year, as well as the number of sires employed by year, is shown in Table 1. Samples from the birth cohorts 2012–2014 (n = 46, 27 and 33 animals) were used in previous studies analyzing microsatellites [2,3]. Samples from 10 stallions were also collected, but mares and fillies could not be sampled [2,3]. The Dülmen wild horse population is not kept in stables and not tamed. The natural behavior of the horses is not disturbed through human interventions, which means that mares and fillies are not accessible for sampling [1,2,3]. This can limit the study, because the genetic diversity of the mares remains unknown and the sample size cannot be increased. Nevertheless, continued sampling over a consecutive period of years may compensate for this restriction on sampling. As the females stay in this herd their whole life and their survival is influenced only through natural selection, samples of colts from 12 consecutive years reflect the genetic diversity of the Dülmen wild horse herd.

A survey on the stallions, their use in the herd of Dülmen wild horses, the number of progenies per season, and the breeding success rate—expressed as the ratio of male auctioneered foals to the duration of the breeding period in days—is given in Table S1.

A total of 337 male Dülmen wild horses, which were sold at auctions, and 10 out of the 14 stallions used in the herd were genotyped with the Illumina GGP Equine Plus Beadchip (Neogen, Lincoln, NE, USA) containing 71.589 single nucleotide polymorphisms (SNPs). Preparation of DNA and genotyping were performed by Neogen (Neogen, Lincoln, NE, USA) using standard Illumina protocols. All further analyses were performed using only autosomal SNPs with genotyping rates > 0.90 for SNPs and animals. The final dataset included 63,123 autosomal SNPs. All animals had genotyping rates > 0.995. The quality of the SNP data was controlled using PLINK v1.9 (www.cog-genomics.org/plink/1.9/, accessed on 2 July 2025), Complete Genomics, Mountain View, CA, USA [28].

### 2.3. Runs of Homozygosity and Fixation Index

We employed the approach for runs of homozygosity (ROH) with overlapping windows as implemented in PLINK v1.9 [28]. All SNPs were retained, which fulfilled the technical requirements (genotyping rates > 0.90 for SNPs and animals). Therefore, SNP genotype data were not pruned for minor allele frequency (MAF) and linkage disequilibrium (LD), following Meyermans et al. [29] and Lencz et al. [30]. The error rates were low in our genotyping data, and an even distribution across the equine genome was guaranteed; therefore, we applied pruning for either MAF or LD. SNPs with a low MAF or deviating from the Hardy–Weinberg equilibrium could be associated with genotyping errors. However, the SNPs on the genotyping platform used have proven to have good technical reproducibility and high polymorphism information content. When SNPs with a low MAF are excluded, ROHs may be missed, because in other horse breeds, polymorphic SNPs are removed from the analysis [29,30]. LD pruning should ensure that detection probabilities of ROH are equally distributed across the genome. Arrays with SNPs not uniformly distributed across the genome may require LD pruning so that recombination distances between SNPs become more similar. We calculated the minimum number of SNPs for a ROH according to Lencz et al. [30] and Purfield et al. [31] with a type I error rate (α) of 0.05, an average SNP heterozygosity of 0.3069, and an average SNP density of 35.478 kb per SNP. We reached the following settings for our data: a minimum SNP density of one SNP per 100 kb, a maximum gap length of 500 kb, a minimum length of homozygous segments of 1923 kb, including 54 or more homozygous SNPs, and a window size of 15 SNPs. One heterozygous SNP genotype and one missing SNP were permitted. The total length of the autosomal chromosomes covered by SNPs was 2,239,477,583 bp. To visualize the ROH length distribution, the ROH segments were categorized into the following length segments: ≤4 Mb, >4–8 Mb, >8–16 Mb, >16–32 Mb, and >32 Mb.

The inbreeding coefficients F_Hat1–3_ were calculated using PLINK v1.9 [28]. F_Hat1_ estimates the variance explained by all autosomal SNPs, F_Hat2_ measures the excess of homozygosity—similarly to the F_IS_ estimate—and F_Hat3_ partitions the genetic variance into each of the 31 autosomes [28].

The genomic inbreeding coefficient (F_ROH_) for each horse was estimated according to McQuillan et al. [32], including all ROH and ROH by length classes in Mb, which inform the timing of inbreeding and comprise F_ROH>4_, F_ROH>8_, F_ROH>16_, and F_ROH>32_, as well as F_ROH>2.25_, F_ROH>3.33_, F_ROH>5_, F_ROH>10_, F_ROH>16.67_, F_ROH>25_, F_ROH>33.33_, F_ROH-2–4_, F_ROH-4–8_, F_ROH-8–16_, and F_ROH-16–32_. Different restrictions on ROH lengths were employed to explore genomic inbreeding for an estimated number of generations in the past. The F_ROH_, F_ROH>2_ F_ROH>4_, F_ROH>8_, F_ROH>16_, and F_ROH>32_ estimates encompass the last 26.0 (derived from the average length of ROH), 25.0, 12.5, 6.25, 3.125, and 1.5625 generations, whereas the estimates for F_ROH>2.25_, F_ROH>3.33_, F_ROH>5_, F_ROH>10_, F_ROH>16.67_, F_ROH>25_, and F_ROH>33.33_ refer to the last 20, 15, 10, 5, 3, 2, and 1.5 generations. Furthermore, F_ROH-2–4_, F_ROH-4–8_, F_ROH-8–16_, and F_ROH-16–32_ include a certain number of generations. These span from 12.5 to 25 (F_ROH-2–4_), 12.5 to 6.25 (F_ROH-4–8_), 3.125 to 6.25 (F_ROH-8–16_), and 1.5625 to 3.125 (F_ROH-16–32_) generations.

The fixation index *F_IS_* (excess of homozygosity) for each individual was calculated using the software SAS, version 9.4 (Statistical Analysis System, Cary, NC, USA, 2025).

### 2.4. Effective Population Size and ROH Islands

Linkage disequilibrium (LD), determined through the squared correlation (r^2^) between pairs of SNPs, was employed to estimate the effective population size (N_e_) with PLINK (www.cog-genomics.org/plink/1.9/, accessed on 3 May 2025) version 1.9 [28]. Between all SNP pairs, r^2^ values were calculated, based on pairwise distances between 1 Kb and 50 Mb. We used distance bins of 10 Kb to 100 to calculate mean r^2^ values. The N_e_ is given by $N_{e}=\frac{\left(1-r^{2}\right)}{\left({4cr}^{2}\right)}$, with c equal to the recombination rate in Morgan units [33], which approximately corresponds to the distance between two SNPs in units of 100 Mb (100 Mb~1 Morgan). The number of generations in the past was determined by $\frac{1}{\left(2c\right)}$. The increase in inbreeding per generation is given by $\Delta F=\frac{1}{{2\;N}_{e}}$ [33].

Calculation of ΔF and N_e_ was also performed using F_ROH_, F_ROH>4_, F_ROH>8_, F_ROH>16_, and F_ROH>32_ and GE inferred from the length of the respective F_ROH_ [34,35]:

$$∆F_{ROH-i}=1-\sqrt[{{GE-ROH}_{i}-1}]{(1-F_{ROH-i}})\;\mathrm{and}\;N_{e-ROH-i}=1/2∆F_{ROH-i}$$

with F_ROH-i_ = F_ROH_, F_ROH>4_, F_ROH>8_, F_ROH>16_, and F_ROH>32_. The parameter GE-ROH_i_ corresponds to 26.0, 12.5, 6.25, 3.125, and 1.5625 generations for F_ROH_, F_ROH>4_, F_ROH>8_, F_ROH>16_, and F_ROH>32_.

ROH islands covered regions that exceeded the 99th percentile of the homozygosity distribution. Consensus ROH were shared by 20%, 25%, 30%, 40%, and 45% of the horses.

Gene annotations for ROH islands and consensus ROH regions were retrieved from the Ensemble genome assembly release 112 of EquCab3 [36]. Molecular functions, biological processes, cellular component, protein class, pathways, and overrepresented genes were identified using PANTHER v19.0 (Protein Analysis Through Evolutionary Relationships) at the Division of Bioinformatics, Department of Preventive Medicine, Keck School of Medicine of USC, University of Southern California, Los Angeles, CA, USA [37,38].

### 2.5. Statistical Analysis of ROH and Breeding Success Rates of Stallions

We employed linear models to analyze ROH by birth cohorts. The genomic inbreeding coefficients were tested for significant effects using the following linear model 1:

*y*_ij_ = µ + birth-year_i_ + e_ij,_ (1)

where y_ij_ = dependent variate with the mean number of ROH, the average ROH length, the total length of ROH, F_IS_, F_ROH_, F_ROH>4,_ F_ROH>8,_ F_ROH>16_, F_ROH>32_, F_ROH>25_, F_ROH>16.67_, F_ROH>10_, F_ROH>5_, F_ROH>3.33_, and F_ROH>2.25_. Birth-year_i_ = is the fixed effect for each of the 12 birth years, with i = 1–12 (2012–2023) and e_ij_ = random error term.

Model 2 is an extension of model 1 and should demonstrate the differences between paternal progeny groups by birth years:

*y*_ijk_ = µ + birth-year × stallion_ij_ + e_ijk,_ (2)

where y_ijk_ = dependent variates, as in model 1. Birth-year × stallion_j_ is the fixed effect of the stallion by birth year, with ij = 1–26 and e_ijk_ = random error term.

Model 3 regarded the linear regression of F_ROH_ or log(1 − F_ROH_) on birth years encoded as 1–12 to visualize the inbreeding trends.

*y*_ij_ = µ + b(F_ROH_) + e_ij_ or *y*_ij_ = µ + b(log(1 − F_ROH_)) + e_ij_ (3)

Model 4 included a linear covariate and a fixed effect (model 4) or fixed and random effects (model 5) to explore the effect of the degree of inbreeding of stallions on the next generation. Models 4 and 5 are as follows:

*y*_ijk_ = µ + birth-year_i_ + b(F_ROH_-stallion)_j_ + e_ijk_, (4)

where y_ijk_ = dependent variates, as in model 1, (F_ROH_-stallion_j_) is the linear covariate for the genomic inbreeding coefficient of the stallion, and b is the linear regression coefficient, with j = 1 and e_ijk_ = random error term.

*y*_ijkl_ = µ + birth-year_i_ + b(F_ROH_-stallion)_j_ + stallion_l_ +e_ijkl_, (5)

where y_ijkl_ = dependent variates, as in model 1. Stallion_l_ = is the random effect for the stallion, with l = 1–10, and e_ijkl_ = random error term.

Instead of the random stallion effect, Model 6 included a random stallion by season effect.

Model 6, which only includes a linear covariate, is as follows:

*y*_ijk_ = µ + bF_ROH_-stallion(stallion)_ij_ + e_ijk_, (6)

where y_ijk_ = dependent variates as in model 1 and bF_ROH_-stallion(stallion)_i_ are the linear covariates for the genomic inbreeding coefficient of the stallions, nested within the respective stallion, with ij = 1–10, and e_ijk_ = random error term.

Seasonal breeding success rates of the stallions were analyzed with the following models. Model 7 included a linear regression on the different F_ROH-stallion_ coefficients, and model 8 on the different F_ROH-stallion_ coefficients, which were split in recent and past generations (F_ROH-stallion>gen-k_ and F_ROH-stallion≤gen-k_) with k = 4, 8, 16, 32, 2.25, 10, 16.67, 25, and 33.33. Model 8 differentiates between more recent and ancestral inbreeding:

*y*_ij_ = µ + bF_ROH-stallioni_ + e_ij_, (7)

y_ijk_ = µ + b_1_F_ROH-stallion>gen-k,i_ + b_2_F_ROH-stallion≤gen-k,j +_ e_ijk_, (8)

where y_ijk_ = dependent variate with the seasonal breeding success rate of the stallions (n = 10), b_1_ and b_2_ are the linear regression coefficients, and e_ijk_ = random error term. All calculations were performed using generalized linear models (GLM) or mixed models (MIXED) of SAS, version 9.4 (Statistical Analysis System, Cary, NC, USA, 2025).

## 3. Results

### 3.1. ROH and Inbreeding Coefficients

The average number of ROH per horse was 27.96 ± 7.692, with an average ROH length of 8.237 Mb ± 2.573 Mb and a combined length of 239.614 ± 12.222 Mb (Table 2).

Average F_ROH_ and F_IS_ estimates were 0.107 ± 0.030 and −0.023 ± 0.064, respectively (Table 3). F_ROH>4_ and F_ROH>8_ moderately decreased to values of 0.093 and 0.076, respectively. F_ROH>3.33_, which corresponds to the inclusion of the last 15 generations, resulted in an estimate of 0.096. F_ROH_-estimates, encompassing the last 10 (F_ROH>5_), 5 (F_ROH>10_), 3 (F_ROH>16.67_), and 2 (F_ROH>25_) generations, reached values of 0.088, 0.068, 0.047, and 0.030, respectively.

F_IS_, F_ROH_, and all other ROH-based inbreeding coefficients, in which smaller ROH were excluded, were highly correlated (Table S2). Correlation coefficients decreased when fragment sizes used for estimation of ROH were more dissimilar. The lowest correlations were observed between F_ROH-2–4_ and F_ROH-16–32_, F_ROH>32_ and F_ROH-2–4_, F_ROH>16_ and F_ROH-2–4_, and F_ROH-2–4_ and F_ROH-4–8_.

Analysis of the cumulative distribution of F_ROH_ by ROH length revealed an increase up to a length of 40 Mb (Supplementary Figure S1). Differences for F_ROH_, average and total ROH length, and number of ROH per horse were not significantly different between birth year cohorts.

A total number of 9424 ROH were identified. In the length categories of 2–4, 4–8, and 8–16 Mb, 39.94%, 22.87%, and 19.12% of all ROH were distributed (Table S3). Lower numbers of ROH, with 3.85% (n = 362) and 3.53% (n = 333) of all ROH, had lengths of <2 and >32 Mb.

The number of ROH, their average lengths, and their combined lengths by the different length classes across each autosomal chromosome are shown in Table S4. On 23 autosomes, there were ROH with a length > 32 Mb. More than 20 F_ROH>32_ were found on autosomes 1, 4 and 10, and even between 15 and 20 F_ROH>32_, they were present on autosomes 2, 3, 6, 14, 15, 17, and 18, respectively. F_ROH-2–4_ were seen on all autosomes.

### 3.2. Effective Population Size

The effective population size N_e_ based on r^2^ values between pairs of neighboring SNPs for the last 200 generations showed a decreasing trend in N_e_. The estimates of N_e_ reached 811 about 200 generations ago. The decline of N_e_ over the generations was 498, 284, 164, 85, 59, and 41 for generations 100, 50, 25, 10, 5, and 1, respectively. The downward trend in N_e_ was nearly constant over the last 100 generations, with about -4.5 per generation, and was slowed down in the last 10 generations. We assumed a generation interval of 15 years because mares are reproducing between 4 to 30 years of age [1], and this herd consists exclusively of female horses. Based on this estimate for the generation interval, 10 generations correspond approximately to 150 years.

Estimates for ΔF_ROH_ and N_e-ROH_ using F_ROH_, F_ROH>4_, F_ROH>8_, F_ROH>16_, and F_ROH>32_ from all horses resulted in values of 0.004516, 0.008411, 0.014987, 0.023434, and 0.034904 for ΔF_ROH_, and 110.71, 59.446, 33.39, 21.34, and 14.33 for N_e-ROH_, respectively.

### 3.3. ROH Islands and Consensus ROH

We identified 10 ROH islands, defined through the 99th percentile threshold, on ECA 1, 4, 5, 7, 9, and 10 (Table 4). The number of identified genes was between 4 on ECA 4 and 61 on ECA 10. Consensus ROH shared by 25% and 30% of the Dülmen wild horses were located on 6 (ECA 1, 4, 5, 7, 9, and 10) and 3 (ECA 1, 5, and 10) equine chromosomes (Table S5). When consensus ROH for 40% of the horses was considered, only two ROH islands were found on equine chromosome 1. The most common ROH with 45% of the horses was on ECA 1, between 110,755,069 and 111,764,486 bp, containing 34 SNPs and 7 genes.

PANTHER gene ontology enrichment analysis revealed four ROH islands on ECA 1, 4, and 10, with significantly overrepresented genes in the category “PANTHER Go-Slim Biological Process” (Table S6). Using the annotation dataset “PANTHER Go-Slim Molecular Function”, genes on ECA 1 and 10 were overrepresented. The ROH island on ECA 1 between 146,406,089 and 148,462,968 bp harbors genes of the phospholipase A2 group IV. The gene *cortactin-binding protein 2* (*CTTNBP2*) is located on the ECA 4 ROH and regulates structure and activity of synapses. In the ECA 10 ROH between 24,145,681 and 26,370,021 bp, there are 14–15 genes overrepresented, including *TNNT1*, which encodes the slow skeletal muscle troponin and links excitation to contraction in skeletal muscle. In addition, genes of the *NRL* family and many *Zinc finger protein* genes reside in this ROH. On the ECA 10 ROH, from 27,584,754 to 28,501,938 bp, only *Zinc finger protein* genes are significantly overrepresented.

### 3.4. Inbreeding by Birth Years

Genomic inbreeding using model 1 was significantly different between birth years (Tables S7 and S8). Differences in genomic inbreeding between stallions within birth years using model 2 were significant for three pairs of stallions, whereas significant differences between years within stallions were only observed for one stallion (Table S9).

The linear regression for F_ROH_ on birth years was slightly negative (−0.0006686 ± 0.0010231, *p*-value = 0.5140) in model 3. With increasing lengths of ROH segments (F_ROH>8_, F_ROH>16_, F_ROH>32_), the negative trend became more apparent with regression coefficients of −0.001077 ± 0.000952, *p*-value = 0.2590; −0.001251 ± 0.000813, *p*-value = 0.1248; and −0.001078 ± 0.000576, *p*-value = 0.0622. The trend for the increase in inbreeding per birth year (ΔF_yr_ = 1 − exp(b)) derived from the regression coefficient b for 1-logF_ROH_ on birth year. It was close to zero (0.00045155) and not significant but positive. The highest estimate was obtained for F_ROH>16_, with an estimate of 0.00101 for ΔF_yr_, but it was also not significant (*p*-value = 0.1885).

Genomic inbreeding parameters were calculated for 10 Dülmen wild horse stallions. The estimates for F_ROH_ ranged between 0.027 and 0.198, with an average value of 0.087 and a standard deviation of 0.050 (Table S10). Employing a model for genomic inbreeding in the male Dülmen wild horses with the effect of birth year for the male progeny and a linear covariate with the F_ROH_ of the 10 genotyped stallions (model 4) did not remove the significant effect of the birth year but had a decreasing effect (−0.7497 ± 0.0279; *p*-value = 0.0076) on the genomic inbreeding coefficients of the male progeny (Table S11). An extended model, including an additional random sire effect (model 5), leads to a non-significant birth year effect. The variance explained by the sire effect on the F_ROH_ of the male progeny was 8.079%. Regarding the random interaction between stallion and season instead of the random sire effect explained 13.040% of the variation in genomic inbreeding F_ROH_ of the male progeny (modified model 5).

Model 6, using a linear covariate with the F_ROH_ of the 10 genotyped stallions nested within the sire of the male progeny, resulted in the lowest residual variance with an estimate of 0.002793. This model captured the heterogeneity of the different sizes of the genomic inbreeding coefficients on the F_ROH_ on their progeny (Table S12). For two stallions, the linear regression coefficients were significant, and for two further stallions, it was close to a *p*-value of 0.05. These four stallions had an important effect on the increase in the F_ROH_ on their progeny above the estimated mean (intercept) value. Among the 10 stallions, these 4 showed genomic inbreeding: two very low, one moderate, and one the highest.

### 3.5. Breeding Success Rates of Stallions and Inbreeding

We compared the seasonal breeding success rates of 10 Dülmen wild horse stallions with their inbreeding parameters based on ROH (Table 5). Pearson correlations and linear regression coefficients with F_IS-stallion_ and F_ROH-stallion_ estimates were negative, but not significantly different from zero. However, the results indicate that inbreeding in the last three generations may be more detrimental for the breeding success of stallions. Simultaneous testing for F_ROH-2–4-stallion_, F_ROH-4–8-stallion_, F_ROH-8–16-stallion_, F_ROH-16–32-stallion_, and F_ROH>32-stallion_ confirmed the negative effects of recent inbreeding on the seasonal breeding success rates with a significant negative linear regression coefficient for F_ROH>32-stallion_ (b = −23.054 ± 9.087; *p*-value = 0.0248); for the other F_ROH-stallion_ parameters, the linear regression coefficients were not significantly different from zero.

We tested whether purging may play a role for the breeding success of stallions using genomic inbreeding coefficients of stallions encompassing either recent or past generations (model 8). Here, we used their ROH length-restricted F_ROH-stallion_ and their difference to F_ROH-stallion_. In this way, we tested inbreeding accumulated within 1.5 (F_ROH>33.33-stallion_) and 20 (F_ROH>2.25-stallion_) generations against inbreeding, which accumulated after 1.5 (F_ROH-stallion_ − F_ROH>33.33-stallion_) and 20 (F_ROH-stallion_ − F_ROH>2.25-stallion_) generations. Considering F_ROH_ for the most recent generations (<1.5 to <3.125), regression coefficients achieve negative estimates up to −17.226, close to a *p*-value of 0.05, whereas the corresponding F_ROH-stallion_, including consecutive past generations, reach positive estimates (Table S13). When analyses were performed for splitting F_ROH-stallion_ at 12.5 or 20 generations, negative regression coefficients were still obtained for inbreeding in the more recent generations, but with increasing positive values in ancestral generations. However, with rather large standard errors, they were estimated for inbreeding associated with the corresponding past generations.

## 4. Discussion

The present study searched for ROH patterns, their temporal origin, and development over 12 consecutive birth cohorts in young male Dülmen wild horses of Merfelderbruch, near Dülmen in Westphalia, Germany. We adhered to the recommendations for identifying ROH; therefore, we did not prune the genotyping data for MAF and LD. By doing so, ROH could be missed, particularly when the analysis included only one population [29,30,31]. The mean and median F_ROH_ were 10.7% and 9.8%. Restricting the ROH segments to sizes, corresponding to ROH that arose within the last 3, 5, and 10 generations with ROH sizes of more than 16.67, 10, and 5 Mb, resulted in mean (median) values of 4.7% (3.7%), 6.8% (5.8%), and 8.8 (7.9%), respectively. Other studies in horse breeds with larger numbers of breeding animals than the Dülmen wild horse population had similar genomic inbreeding coefficients, including Belgian draught horses, who showed an average F_ROH_ of 10.1% [20], European warmblood with an average F_ROH_ of 10.99%, Noriker and Norik of Muran with a mean F_ROH_ of 10% and 11% [15], and Rhenish coldblood with an average F_ROH_ of 9.9%, respectively [27]. In contrast, in Polish coldblood horses, genomic inbreeding was lower, with an estimate of 6.1% [9]. Italian heavy draught [16] and Friesian horses reached higher estimates, with 15.4% and 22.3% [18,39], similarly to Polish Konik, which reached 15.96% [10]. Also, in the Franches–Montagnes and the old-type of Franches–Montagnes horses, F_ROH_ were higher, with estimates of 12.04% and 13.37%, respectively [17]. Austrian and Italian Haflinger horses reached mean F_ROH_ values of 12.6% and 14.1%, respectively [13]. Arabian, Shagya Arabian, and Thoroughbred had even higher mean F_ROH_, 15.65%, 13.69%, and 19.12%, respectively [17]. These comparisons lead us to believe that horse breeds with larger populations that are subject to breeding programs have accumulated similar or even more ROH when compared to the Dülmen wild horse population with its closed mare population.

We classified F_ROH_ by their lengths of ROH segments to gain insights into the degree of inbreeding in the most recent and corresponding earlier generations. The results suggest that genomic inbreeding has increased steadily over the last 20 generations. There were no indications of periods of a sharp increase in genomic inbreeding. Estimates on LD agreed with the results from ROH. The reason for this may be that there were only natural selection and no breeding program with a strong selection of future stallions and mares. Assuming a generation interval of 15 years [1,2,3], we may date the fencing of the wild horses in 1856 to approximately 10–11 generations ago. A steeper increase in genomic inbreeding around this time was not obvious. Nevertheless, the effective population size decreased from 85 to 41 within the last 10 generations. Compared to much larger horse populations, such as endangered coldblood horse breeds with Ne estimates between 46 and 59 [16,27], the effective population size of the Dülmen wild horses remained reasonable.

This contrasts with breeding populations, where an increase in inbreeding with the foundation of a studbook can be observed, e.g., in Rhenish German Draught horses [27]. In line with LD estimates for increase in inbreeding and F_ROH_ estimated for different ROH segment lengths, the trend of F_ROH_ across birth cohorts was close to zero. However, we could observe significant differences for F_ROH_ between the different 12 birth years. A reason for this may be the stallions causing a subclustering of the Dülmen wild horse herd [3]. The stallions showed a wide variation in their genomic inbreeding as well as large differences in their influence on genomic inbreeding in their offspring. The mating design did not allow us to separate the effects of genomic inbreeding due to stallions from the effects of birth cohort, as stallions and birth years were partly confounded. Thus, the stallion effects and the effect of the stallions’ F_ROH_ may be partly influenced by the mating behavior of the mares, leading to non-random breeding. After correction for the F_ROH_ of the individual stallions, the effect of the birth year was no longer significant. On the other hand, regression on the F_ROH_ of individual stallions was significant and related in nearly all 10 stallions, with an increase in inbreeding in their progeny. Thus, relatedness among stallions and mares may be important for the future development of effective population size and genomic inbreeding. To reduce increase in inbreeding in future generations, genotyping of potential stallions on a high-density SNP array should be performed before breeding to estimate their genomic kinship matrices and genomic inbreeding coefficients. The most valuable tool to minimize future increase in inbreeding should be achieved through the estimation of the expected inbreeding load of the future progeny [39,40]. This approach uses genome region-specific haplotypes to derive haplotype diversity in the next generation [39]. A major limitation of this approach is that the genotypes of the mares cannot be provided. Instead of the mares’ genotypes, we could use the data from all yearlings as substitutes for the mares. This may be a useful support for breeding decisions on future stallions besides phenotypic criteria, including body characteristics, temperament, and behavior. The only way to influence the development of inbreeding in future generations is through the selection of stallions, as herd size is limited by the available land area, and selection on the mare side is not possible through human intervention. Our analyses suggest that metrics related to genomic architecture are much more suitable for predicting inbreeding effects in future offspring than either pedigree-based measures or metrics averaged across the autosomal genome.

A further issue of genomic inbreeding emerged when analyzing the breeding success of stallions, expressed by the ratio of the number of male yearlings and the duration of the breeding period in days. Higher genomic inbreeding of stallions was associated with decreasing breeding success and a lower rate of progeny per season. Particularly, inbreeding in very recent generations seems to be detrimental to the fertility of stallions. Therefore, the degree of genomic inbreeding should also be regarded in the breeding decisions for future stallions. While purging effects may have only minor effects, they may be present.

ROH islands were found on horse chromosomes 1, 4, 5, 7, 9, and 10. Two of the ten ROH islands (ECA 9 at 35–36 and 10 at 24–26 Mb) were also identified in domesticated horse breeds, including the Polish draught horse breeds Sokolski and Sztumski, the Hucul horse, and Rhenish German draught horse [9,27]. However, Dülmen wild horses did not share ROH islands with European warmblood, Arabian, Shagya Arabian, English Thoroughbred, and Franches-Montagnes horses [17].

The PANTHER overrepresentation tests revealed four ROH islands on ECA 1, 4, and 10, with genes responsible for phospholipid catabolism, skeletal muscle and synapse function, inflammation response, immune system, reproduction, regulation of transcription, and many cellular processes through *zinc finger protein* genes.

Overrepresented genes on ECA 10 include members of the *NLR* gene family with pyrin domain, such as *NLRP2*, *4*, *5*, and *13*. Genes of the *NLR* family were associated with infertility in humans, mice, cattle, and pigs [41,42,43,44,45].

Within the ROH island on ECA 10, many *zinc finger protein* genes were overrepresented. Zinc finger proteins (ZNFs) are involved in a wide range of cellular processes and have key roles in the development and differentiation of several tissues. In addition, ZNFs may act as tumor-suppressor genes and oncogenes, and they have an impact on neurodegenerative and skin diseases and diabetes in humans [46,47,48]. Due to the manifold roles of ZNFs in cellular processes, linking to specific traits for Dülmen wild horses does not seem possible. ZNFs might be important for survival under harsh environmental conditions, but this needs to be proven by further analysis. Further research should investigate ROH islands using whole genome sequencing data, their possible impact on molecular functions, pathways, and recessive lethal variants, as well as their ancestral origin.

## 5. Conclusions

The results of this study demonstrate the maintenance of the genetic diversity in a small horse population over more than 160 years under harsh environmental conditions as an independent population. The effective population size declined smoothly without steep declines. The identified ROH islands were not indicative for a few strong signatures of selection towards a few phenotypic characteristics. Potential stallions should be screened for their degree of genomic kinship and inbreeding, and the expected inbreeding load of the future progeny should be estimated. Reproductive efficiency is expected to decrease with increasing genomic inbreeding, particularly, due to inbreeding in recent generations. Thus, F_ROH_ analyses must consider the lengths of the ROH segments to differentiate between the temporal origin of genomic inbreeding. The present study shows the importance of using genomic data for the population of Dülmen wild horses as pedigree data are not available and the positive or negative contributions of stallions to the genetic diversity in this herd are unpredictable without genomic data. The results of this study highlight the urgent need to monitor genetic diversity in candidates selected as future breeding stallions and in male yearlings captured annually as proxies for this offspring generation using genome-wide genotype data to support the long-term conservation of this unique horse population. Further research could also analyze potential recessive lethal variants based on data from whole-genome sequencing.

## Figures and Tables

**Table 1:** Number of stallions, estimated number of mares (females > 2 years old), genotyped male horses, and number of males at auctions.

Year of Siring	Birth Year	Year of Auction	Stallions	Mares	Males Genotyped	Males at Auctions
2011	2012	2013	2	238	25	46
2012	2013	2014	3	371	12	27
2013	2014	2015	2	389	15	33
2014	2015	2016	2	389	25	45
2015	2016	2017	2	429	28	28
2016	2017	2018	2	430	35	35
2017	2018	2019	2	389	24	24
2018	2019	2020	2	374	31	31
2019	2020	2021	2	368	41	41
2020	2021	2022	2	404	37	37
2021	2022	2023	2	374	34	34
2022	2023	2024	3	376	30	30
Total			14		337	411

**Table 2:** Mean, standard deviation (SD), minimum (Min), and maximum (Max) of the average number of ROH, average ROH length, and combined length of ROH in all genotyped male Dülmen wild horses (n = 337).

ROH Items	Mean	SD	95% CI	75% CI	Min	Max
Average number of ROH	27.96	7.692	16–41	22–33	7	52
Average ROH length (Mb)	8.237	2.573	4.488–13.105	6.610–9.773	2.884	22.208
Combined ROH length (Mb)	239.614	120.222	82.586–471.985	151.183–306.979	20.188	777.280

**Table 3:** Means, medians, modes, standard deviations (SD), and 95% and 75% confidence intervals (CI) of genome-based inbreeding coefficients for all genotyped male Dülmen wild horses (n = 337).

Inbreeding Coefficients	Mean	SD	Median	Mode	95% CI	75% CI
F_IS_	−0.023	0.064	−0.031	−0.089	−0.105–0.106	−0.065–0.012
F_HOM_	0.694	0.019	0.691	0.682	0.668–0.742	0.680–0.703
F_ROH_	0.107	0.030	0.098	-	0.037–0.211	0.068–0.137
F_HAT1_	−0.121	0.104	−0.123	-	−0.270–0.051	−0.190–0.060
F_HAT2_	−0.018	0.098	−0.007	-	−0.165–0.121	−0.073–0.044
F_HAT3_	−0.018	0.045	−0.022	-	−0.080–0.068	−0.048–0.005
F_ROH>4_	0.093	0.054	0.083	-	0.023–0.197	0.053–0.121
F_ROH>8_	0.076	0.050	0.066	-	0.014–0.178	0.040–0.104
F_ROH>16_	0.049	0.042	0.038	-	0.000–0.135	0.000–0.071
F_ROH>32_	0.020	0.029	0.014	-	0.000–0.084	0.000–0.030
F_ROH>2.25_	0.103	0.054	0.093	-	0.033–0.207	0.063–0.133
F_ROH>3.33_	0.096	0.054	0.086	-	0.027–0.203	0.056–0.124
F_ROH>5_	0.088	0.052	0.079	-	0.022–0.187	0.051–0.117
F_ROH>10_	0.068	0.049	0.058	-	0.007–0.168	0.034–0.093
F_ROH>16.67_	0.047	0.042	0.037		0.000–0.134	0.018–0.067
F_ROH>25_	0.030	0.035	0.016		0.000–0.097	0.000–0.045
F_ROH>33.33_	0.019	0.029	0.000		0.000–0.081	0.000–0.025
F_ROH-2–4_	0.013	0.004	0.013	-	0.007–0.021	0.011–0.016
F_ROH-4–8_	0.016	0.008	0.016	-	0.005–0.030	0.010–0.022
F_ROH-8–16_	0.027	0.015	0.026	-	0.004–0.055	0.016–0.036
F_ROH-16–32_	0.029	0.021	0.026	-	0.000–0.070	0.014–0.042

**Table 4:** ROH islands with their chromosomal location (ECA), start and end position in bp, and number of SNPs and genes located within the ROH islands of the male Dülmen wild horses (n = 337).

ECA	Start Position (bp)	End Position (bp)	Number of SNPs	Number of Genes
1	109,383,207	112,700,094	75	13
1	146,406,089	148,462,968	26	37
4	74,950,902	75,460,147	42	4
5	44,433,546	47,256,602	78	32
5	48,223,003	51,341,691	70	29
7	49,803,523	51,698,577	63	13
9	35,261,007	36,940,286	39	7
10	24,145,681	26,370,021	62	61
10	27,584,754	28,501,938	29	14
10	68,150,825	72,903,290	142	16

**Table 5:** Correlation and linear regression coefficients with their standard errors (b_SE_) and *p*-values of genomic inbreeding parameters of stallions (F_IS-stallion_, F_ROH-stallion_) in relation to their seasonal breeding success rates in male Dülmen wild horses (n = 19) using model 7.

Inbreeding	Pearson Correlation	Regression Cofficient (b)	b_SE_	*p*-Value
F_IS-stallion_	−0.293	−1.225	0.971	0.2240
F_ROH-stallion_	−0.282	−1.422	1.173	0.2421
F_ROH>4-stallion_	−0.294	−1.563	1.231	0.2210
F_ROH>8-stallion_	−0.284	−1.626	1.330	0.2384
F_ROH>16-stallion_	−0.335	−2.814	1.920	0.1609
F_ROH>32-stallion_	−0.427	−8.915	4.574	0.0680
F_ROH>10-stallion_	−0.265	−1.700	1.498	0.2725
F_ROH>16.67-stallion_	−0.331	−2.779	1.924	0.1667
F_ROH>25-stallion_	−0.320	−4.135	2.965	0.1811
F_ROH>33.33-stallion_	−0.427	−8.915	4.574	0.0680

## Data Availability

Restrictions apply to the availability of these data. The samples were obtained from Dülmen wild horses in the Merfelder Bruch with allowance of Rudolph Herzog von Croÿ and are available from the authors at a reasonable request and with the permission of the horse owner.

## Supplementary Materials

The following supporting information can be downloaded at https://www.mdpi.com/article/10.3390/genes16091054/s1: Figure S1: Cumulative distribution of F_ROH_ in 337 male Dülmen wild horses by the mean length of ROH in Mb (on the *x*-axis); Table S1: Stallions used in the Dülmen wild horse population of the Merfelder Bruch with their sires, dates and duration of the covering period, number of male progeny genotyped and at auction as well as the breeding success rate per stallion and the timespan of the breeding period in days (BSR = number of male progeny at auction divided by duration of covering period in days); Table S2: Pearson correlation coefficients for selected pairs of inbreeding coefficients for the male Dülmen wild horses (n = 337); Table S3: Number and average ROH lengths (Mb) with their standard deviations (SD), minima (Min), and maxima (Max) by length classes of the male Dülmen wild horses (n = 337); Table S4: Distribution of Runs of Homozygosity (ROH) with number of ROH, mean length in Mb, standard deviation, minimum and maximum of ROH length as well as combined length by autosomal chromosomes and length categories in Dülmen wild horses (n = 337). Table S5: Consensus ROH shared by at least 25%, 30%, 40% and 45% of the horses; Table S6: PANTHER gene annotation and enrichment analysis of ROH islands; Table S7: Least-square mean estimates (LSM) with their standard errors (SE) for F_IS_, F_ROH_, F_ROH>4_, F_ROH>8_, F_ROH>16_, and F_ROH>32_ by birth years for 337 male Dülmen wild horses using model 1; Table S8: Least-square mean estimates (LSM) with their standard errors (SE) for F_ROH>25_, F_ROH>16.67_, F_ROH>10_, F_ROH>5_, F_ROH>3.33_, and F_ROH>2.25_, by birth years for 337 male Dülmen wild horses using model 1; Table S9: Least-square mean estimates (LSM) with their standard errors (SE) for F_ROH_ by stallion and birth year for 337 male Dülmen wild horses using a model regarding stallion by birth year of male progeny as a fixed effect (model 2); Table S10: Means, standard deviations, minima and maxima for genomic inbreeding in the 10 genotyped Dülmen wild horse stallions; Table S11: Least-square mean estimates (LSM) with their standard errors (SE) for F_ROH_ by birth years for 256 male Dülmen wild horses using a model regarding birth year of male progeny as a fixed effect and the linear covariate genomic inbreeding of sires (model 4) and in addition, with a model regarding birth year of male progeny as a fixed effect, the linear covariate genomic inbreeding of sires and a random sire effect (model 5) and compared with LSM for birth year from model 1 including 256 male Dülmen wild horses; Table S12: Estimates (LSM) with their standard errors (SE) for F_ROH_ of 256 male Dülmen wild horses using a model regarding the linear covariate genomic inbreeding of stallions (F_ROH_-stallion) within stallion (model 6); Table S13: Linear regression coefficients with their standard errors (b_SE_) and *p*-values of genomic inbreeding parameters of stallions on their seasonal breeding success rates in male Dülmen wild horses (n = 19) using simultaneously genomic inbreeding coefficients estimated either from recent or past generations using model 8.
