# Supplementary material for: Insights into Genomic Patterns of Homozygosity in the Endangered Dülmen Wild Horse Population
Source: Genes (Basel). 2025 Sep 8;16(9):1054. doi: 10.3390/genes16091054 (PMC12469691; doi:10.3390/genes16091054)
Supplement: Supplementary file 1 [file genes-16-01054-s001.zip › Figure S1.pdf]

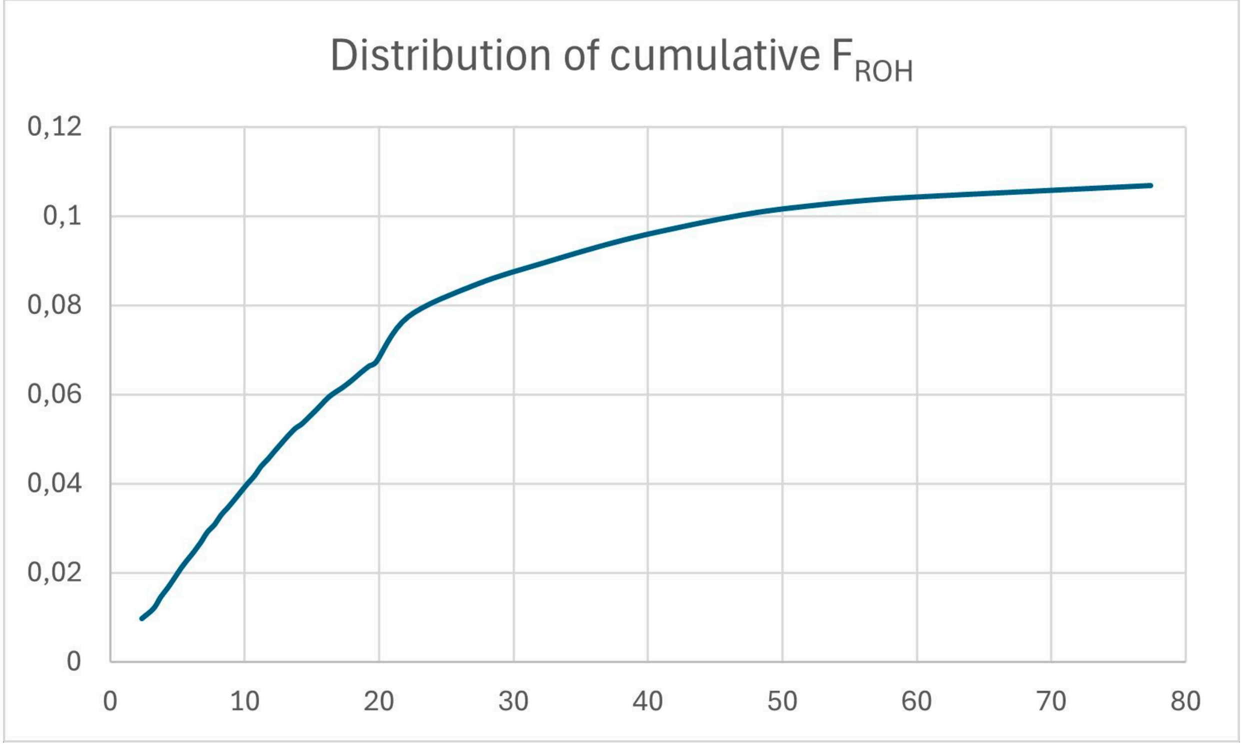

Figure S1. Cumulative distribution of F_{ROH} in 337 male Dülmen wild horses by the mean length of ROH in Mb (on the x-axis).
