# Supplementary material for: Insights into Genomic Patterns of Homozygosity in the Endangered Dülmen Wild Horse Population
Source: Genes (Basel). 2025 Sep 8;16(9):1054. doi: 10.3390/genes16091054 (PMC12469691; doi:10.3390/genes16091054)
Supplement: Supplementary file 1 [file genes-16-01054-s001.zip › Table S1.pdf]

Table S1. Stallions used in the Dülmen wild horse population of the Merfelder Bruch with their sires, dates and duration of the covering period, number of male progeny genotyped and at auction as well as the breeding success rate per stallion and the timespan of the breeding period in days (BSR = number of male progeny at auction divided by duration of covering period in days).

Stallion	Sire	Covering period			Male progeny		BSR
		Year	Dates	Days	Genotyped	At auction	
Finley 58	Sahib	2011	11.04.-20.05.	39	12	14	0.35897
Duncan	Sahib	2011	11.04.-20.05.	39	13	32	0.82051
		2012	23.04.-14.05.	22	8	19	0.86364
Fugato 34	Sahib	2012	04.05.-14.05.	11	1	3	0.27273
		2013	15.04.-16.05.	31	10	18	0.58065
Darius 63	Sahib	2012	23.04.-04.05.	12	3	5	0.41667
Varus	Sir	2013	15.04.-16.05.	31	5	15	0.48387
Faruk 86	Hurmak	2014	14.04.-19.05.	36	20	25	0.69444
		2015	13.04.-15.05.	33	20	20	0.60606
		2016	14.04.-17.05.	34	21	21	0.61765
Agamemnon 58	Nando	2014	14.04.-19.05.	36	5	6	0.17361
		2015	13.04.-15.05.	33	8	8	0.24242
		2016	14.04.-17.05.	34	14	14	0.41176
Abba 56	Nando	2017	12.04.-15.05.	34	19	19	0.55882
		2018	10.04.-15.05.	36	24	24	0.66667
Donatello 13	Duncan	2017	12.04.-15.05.	34	5	5	0.14706
Dorian 23	Duncan	2018	10.04.-15.05.	36	7	7	0.19444
		2019	15.04.-13.05.	28	10	10	0.35714
		2020	15.04.-13.05.	28	9	9	0.32143
Aramis	Nando	2019	15.04.-13.05.	28	31	31	1.10714
		2020	15.04.-13.05.	28	28	28	1.00000

		2021	19.04.-17.05.	28	26	26	0.92857
Valentino 24	Varus	2021	19.04.-17.05.	28	8	8	0.28571
		2022	19.04.-22.04.	3	2	2	0.66667
Vincent	Varus	2022	25.04.-13.05.	18	17	17	0.94444
Salerno	Faruk 86	2022	19.04.-13.05.	24	11	11	0.45833
