# Supplementary material for: Insights into Genomic Patterns of Homozygosity in the Endangered Dülmen Wild Horse Population
Source: Genes (Basel). 2025 Sep 8;16(9):1054. doi: 10.3390/genes16091054 (PMC12469691; doi:10.3390/genes16091054)
Supplement: Supplementary file 1 [file genes-16-01054-s001.zip › Table S11.pdf]

Table S11. Least-square mean estimates (LSM) with their standard errors (SE) for F_{ROH} by birth years for 256 male Dülmen wild horses using a model regarding birth year of male progeny as a fixed effect and the linear covariate genomic inbreeding of sires (model 4) and in addition, with a model regarding birth year of male progeny as a fixed effect, the linear covariate genomic inbreeding of sires and a random sire effect (model 5) and compared with LSM for birth year from model 1 including 256 male Dülmen wild horses.

Birth year	Model 1 for F_{ROH}		Model 4 for F_{ROH}		Model 5 for F_{ROH}	
	LSM	SE	LSM	SE	LSM	SE
2012	0.092	0.011	0.115	0.013	0.113	0.018
2013	0.095	0.016	0.096	0.015	0.096	0.018
2014	0.130	0.017	0.091	0.022	0.098	0.028
2015	0.112	0.024	0.201	0.040	0.184	0.053
2016	0.144	0.019	0.233	0.038	0.216	0.051
2017	0.141	0.015	0.229	0.036	0.212	0.050
2018	0.120	0.013	0.120	0.012	0.113	0.018
2019	0.126	0.010	0.119	0.010	0.113	0.015
2020	0.106	0.009	0.082	0.012	0.098	0.017
2021	0.112	0.009	0.089	0.013	0.104	0.018
2022	0.087	0.011	0.065	0.013	0.087	0.019
2023	0.096	0.010	0.097	0.010	0.097	0.015
