# Supplementary material for: Insights into Genomic Patterns of Homozygosity in the Endangered Dülmen Wild Horse Population
Source: Genes (Basel). 2025 Sep 8;16(9):1054. doi: 10.3390/genes16091054 (PMC12469691; doi:10.3390/genes16091054)
Supplement: Supplementary file 1 [file genes-16-01054-s001.zip › Table S12.pdf]

Table S12. Estimates (LSM) with their standard errors (SE) for F_{ROH} of 256 male Dülmen wild horses using a model regarding the linear covariate genomic inbreeding of stallions (F_{ROH}-stallion) within stallion (model 6).

Effect	Model 6 for F_{ROH}-stallion		
	Estimate	SE	p-value
Intercept	0.093	0.013	<0.0001
F_{ROH}-stallion (Abba 56)	0.360	0.191	0.0607
F_{ROH}-stallion (Agamemnon 58)	0.221	0.083	0.0080
F_{ROH}-stallion (Aramis)	0.028	0.282	0.9206
F_{ROH}-stallion (Darius 63)	0.169	0.334	0.6122
F_{ROH}-stallion (Dorian 23)	1.331	0.428	0.0021
F_{ROH}-stallion (Duncan)	0.111	0.215	0.6054
F_{ROH}-stallion (Finley)	-0.158	0.142	0.2680
F_{ROH}-stallion (Fugato 34)	1.387	0.767	0.0716
F_{ROH}-stallion (Salerno)	0.114	0.273	0.6752
F_{ROH}-stallion (Vincent)	0	-	-
