# Supplementary material for: Insights into Genomic Patterns of Homozygosity in the Endangered Dülmen Wild Horse Population
Source: Genes (Basel). 2025 Sep 8;16(9):1054. doi: 10.3390/genes16091054 (PMC12469691; doi:10.3390/genes16091054)
Supplement: Supplementary file 1 [file genes-16-01054-s001.zip › Table S13.pdf]

Table S13. Linear **r**egression coefficients with their standard errors (b_{SE}) and p-values of genomic inbreeding parameters of stallions on their seasonal breeding success rates in male Dülmen wild horses (n=19) using simultaneously genomic inbreeding coefficients estimated either from recent or past generations using model 8.

**Inbreeding**	**Generations included by $F_{ROH\text{-}stallion}$**	**Regression coefficient (b)**	**b_{SE}**	**P-value**
$F_{ROH>4\text{-}stallion}$	<12.5	-2.117	1.492	0.1751
$F_{ROH\leq4\text{-}stallion}$	≥12.5	11.039	16.234	0.5062
$F_{ROH>8\text{-}stallion}$	<6.25	-1.600	1.708	0.3629
$F_{ROH\leq8\text{-}stallion}$	≥6.25	-0.209	8.308	0.9802
$F_{ROH>16\text{-}stallion}$	<3.125	-3.597	2.763	0.2115
$F_{ROH\leq16\text{-}stallion}$	≥3.125	1.386	3.434	0.6919
$F_{ROH>32\text{-}stallion}$	<1.5625	-17.226	8.480	0.0592
$F_{ROH\leq32\text{-}stallion}$	≥1.5625	3.018	2.603	0.2634
$F_{ROH>2.25\text{-}stallion}$	<20.0	-2.162	1.562	0.1854
$F_{ROH\leq2.25\text{-}stallion}$	≥20.0	62.068	86.849	0.4851
$F_{ROH>10\text{-}stallion}$	<5.0	-0.702	2.441	0.7774
$F_{ROH\leq10\text{-}stallion}$	≥5.0	-3.924	7.475	0.6068
$F_{ROH>16.67\text{-}stallion}$	<3.0	-3.519	2.810	0.2285
$F_{ROH\leq16.67\text{-}stallion}$	≥3.0	1.301	3.513	0.7161
$F_{ROH>25\text{-}stallion}$	<2.0	-5.447	5.924	0.3715
$F_{ROH\leq25\text{-}stallion}$	≥2.0	0.928	3.591	0.7994
$F_{ROH>33.33\text{-}stallion}$	<1.5	-17.226	8.480	0.0592
$F_{ROH\leq33.33\text{-}stallion}$	≥1.5	3.018	2.603	0.2634
