# Supplementary material for: Insights into Genomic Patterns of Homozygosity in the Endangered Dülmen Wild Horse Population
Source: Genes (Basel). 2025 Sep 8;16(9):1054. doi: 10.3390/genes16091054 (PMC12469691; doi:10.3390/genes16091054)
Supplement: Supplementary file 1 [file genes-16-01054-s001.zip › Tables S2 and S3.pdf]

Table S2. Pearson correlation coefficients for selected pairs of inbreeding coefficients for the male Dülmen wild horses (n = 337).

	F_{ROH}	$F_{ROH>4}$	$F_{ROH>8}$	$F_{ROH>16}$	$F_{ROH>32}$	$F_{ROH-2-4}$	$F_{ROH-4-8}$	$F_{ROH-8-16}$	$F_{ROH-16-32}$
F_{IS}	0.970	0.968	0.958	0.917	0.807	0.134	0.450	0.623	0.725
F_{ROH}		0.997	0.986	0.941	0.830	0.142	0.469	0.651	0.740
$F_{ROH>4}$			0.990	0.946	0.834	0.062	0.469	0.647	0.746
$F_{ROH>8}$				0.962	0.850	0.057	0.340	0.636	0.756
$F_{ROH>16}$					0.894	0.029	0.285	0.402	0.771
$F_{ROH>32}$						0.025	0.238	0.326	0.403
$F_{ROH-2-4}$							0.055	0.111	0.023
$F_{ROH-4-8}$								0.334	0.245
$F_{ROH-8-16}$									0.356

Table S3. Number and average ROH lengths (Mb) with their standard deviations (SD), minima (Min), and maxima (Max) by length classes of the male Dülmen wild horses (n = 337).

Length Classes in Mb	Number of ROH	Percent	Mean Length	SD	Min	Max
0-2	362	3.84	1.961	0.224	1.923	1.999
2–4	3,764	39.94	2.704	0. 558	2.000	4.000
4–8	2,155	22.87	5.744	1.136	4.000	7.999
8–16	1,802	19.12	11.318	2.245	8.008	16.000
16–32	1,008	10.70	21.980	4.335	16.002	31.954
>32	333	3.53	44.842	12.037	32.033	131.719
