# Supplementary material for: Insights into Genomic Patterns of Homozygosity in the Endangered Dülmen Wild Horse Population
Source: Genes (Basel). 2025 Sep 8;16(9):1054. doi: 10.3390/genes16091054 (PMC12469691; doi:10.3390/genes16091054)
Supplement: Supplementary file 1 [file genes-16-01054-s001.zip › Tables S7-S9.pdf]

Table S7. Least-square mean estimates (LSM) with their standard errors (SE) for F_{IS}, F_{ROH}, $F_{ROH>4}$, $F_{ROH>8}$, $F_{ROH>16}$, and $F_{ROH>32}$ by birth years for 337 male Dülmen wild horses using model 1.

Birth year	F_{IS}		F_{ROH}		$F_{ROH>4}$		$F_{ROH>8}$		$F_{ROH>16}$		$F_{ROH>32}$	
	LSM	SE	LSM	SE	LSM	SE	LSM	SE	LSM	SE	LSM	SE
2012	-0.0390	0.012	0.092	0.010	0.079	0.010	0.066	0.010	0.043	0.008	0.016	0.005
2013	-0.0400	0.018	0.095	0.015	0.083	0.015	0.071	0.014	0.048	0.012	0.020	0.008
2014	-0.0191	0.016	0.105	0.013	0.093	0.013	0.077	0.012	0.053	0.011	0.024	0.007
2015	-0.0511	0.012	0.083	0.010	0.068	0.010	0.054	0.010	0.035	0.008	0.013	0.006
2016	0.0076	0.012	0.125	0.010	0.109	0.010	0.091	0.009	0.059	0.008	0.022	0.005
2017	0.0152	0.010	0.134	0.009	0.118	0.009	0.100	0.008	0.065	0.007	0.032	0.005
2018	-0.0131	0.012	0.116	0.011	0.102	0.010	0.084	0.010	0.057	0.008	0.025	0.006
2019	-0.0015	0.011	0.126	0.009	0.110	0.009	0.092	0.009	0.062	0.007	0.030	0.005
2020	-0.0283	0.010	0.106	0.008	0.093	0.008	0.077	0.008	0.049	0.006	0.018	0.004
2021	-0.0222	0.010	0.112	0.009	0.099	0.008	0.082	0.008	0.055	0.007	0.023	0.005
2022	-0.0553	0.010	0.081	0.009	0.066	0.009	0.052	0.008	0.030	0.007	0.010	0.005
2023	-0.0372	0.011	0.094	0.009	0.078	0.009	0.061	0.009	0.032	0.008	0.004	0.005

Table S8. Least-square mean estimates (LSM) with their standard errors (SE) for $F_{ROH>25}$, $F_{ROH>16.67}$, $F_{ROH>10}$, $F_{ROH>5}$, $F_{ROH>3.33}$, and $F_{ROH>2.25}$, by birth years for 337 male Dülmen wild horses using model 1.

Birth year	$F_{ROH>25}$		$F_{ROH>16.67}$		$F_{ROH>10}$		$F_{ROH>5}$		$F_{ROH>3.33}$		$F_{ROH>2.25}$	
	LSM	SE	LSM	SE	LSM	SE	LSM	SE	LSM	SE	LSM	SE
2012	0.029	0.007	0.041	0.008	0.060	0.009	0.076	0.010	0.081	0.010	0.089	0.010
2013	0.031	0.010	0.045	0.011	0.064	0.014	0.079	0.015	0.085	0.015	0.091	0.015
2014	0.033	0.009	0.052	0.010	0.070	0.012	0.088	0.013	0.095	0.013	0.101	0.013
2015	0.021	0.007	0.033	0.008	0.048	0.009	0.065	0.010	0.072	0.010	0.078	0.010
2016	0.034	0.007	0.057	0.007	0.083	0.009	0.104	0.010	0.113	0.010	0.120	0.010
2017	0.042	0.006	0.063	0.006	0.090	0.008	0.114	0.009	0.122	0.009	0.130	0.009
2018	0.037	0.007	0.056	0.008	0.077	0.010	0.098	0.010	0.106	0.011	0.113	0.011
2019	0.042	0.006	0.059	0.007	0.085	0.008	0.106	0.009	0.114	0.009	0.122	0.009
2020	0.025	0.005	0.047	0.006	0.070	0.007	0.088	0.008	0.095	0.008	0.102	0.008
2021	0.034	0.006	0.054	0.006	0.075	0.008	0.095	0.008	0.102	0.008	0.109	0.009
2022	0.016	0.006	0.028	0.006	0.045	0.008	0.062	0.009	0.069	0.009	0.077	0.009
2023	0.013	0.006	0.030	0.007	0.053	0.009	0.073	0.009	0.083	0.009	0.090	0.009

Table S9. Least-square mean estimates (LSM) with their standard errors (SE) for F_{ROH} by stallion and birth year for 337 male Dülmen wild horses using a model regarding stallion by birth year of male progeny as a fixed effect (model 2).

Stallion	Birth year	F_{ROH}-LSM	F_{ROH}-SE
Abba 56	2018	0.119	0.012
Abba 56	2019	0.123	0.011
Agamemnon 58	2015	0.112	0.022
Agamemnon 58	2016	0.144	0.018
Agamemnon 58	2017	0.141	0.013
Aramis	2020	0.095*	0.009
Aramis	2021	0.099*	0.009
Aramis	2022	0.087	0.010
Donatello 13	2018	0.106	0.022
Darius 63	2013	0.109	0.029
Dorian 23	2019	0.137	0.019
Dorian 23	2020	0.139	0.016
Dorian 23	2021	0.155	0.017
Duncan	2012	0.112*	0.014
Duncan	2013	0.085	0.018
Faruk 86	2015	0.076**	0.011
Faruk 86	2016	0.117	0.011
Faruk 86	2017	0.129	0.011
Finley	2012	0.071	0.015
Fugato 34	2013	0.130	0.050
Fugato 34	2014	0.130*	0.016
Salerno	2023	0.101	0.015
Valentino 24	2022	0.062	0.018
Valentino 24	2023	0.072	0.036
Varus	2014	0.056	0.022
Vincent	2023	0.093	0.012

**: Significant differences within stallions, but between birth years: Faruk 86 in 2015 versus Faruk 86 in 2016 and 2017 (p-values < 0.01)
Significant differences between stallions within birth years: 2012 for Duncan vs. Finley (p-value < 0.05), 2014 for Fugato 34 vs. Varus (p-value < 0.001), 2020 and 2021 for Aramis vs. Dorian 23 (p-value < 0.05 and p-value < 0.01). The fixed effect of birth year was not significant (p-value = 0.8024) and thus*, omitted in this model.
